# The impact of ageing on adults with cerebral palsy: the results of a national online survey

**DOI:** 10.3399/BJGPO.2023.0028

**Published:** 2023-12-13

**Authors:** Brian G Bell, Sonali Shah, Neil Coulson, Janice McLaughlin, Pip Logan, Richard Luke, Anthony J Avery

**Affiliations:** 1 School of Medicine, University of Nottingham, Nottingham, UK; 2 School of Geography, Politics, and Sociology, Newcastle University, Newcastle upon Tyne, UK; 3 Scope Here East Press Centre, London, UK

**Keywords:** cerebral palsy, general practice, ageing, adult, mobility, fatigue, pain

## Abstract

**Background:**

Cerebral palsy (CP) is one of the most common neurological disorders in children and results in lifelong physical impairments. Adults with CP have approximately the same life expectancy as their non-disabled peers, so helping them to stay healthy throughout the life course will have long-term cost benefits via reductions in hospital admissions, long-term care, and unemployment rates.

**Aim:**

To explore how adults with CP experience ageing.

**Design & setting:**

National online survey given to adults with CP in the UK.

**Method:**

The participants were adults with CP. Items for the online survey were taken from existing self-report measures, with additional items developed for the survey. Several domains of functioning were assessed including mobility, dexterity, fatigue, pain, speech, mental health, swallowing and health maintenance/self-care as well as healthcare usage. Data were analysed using χ^2^ to examine the relationships between the demographic variables and the survey responses.

**Results:**

The survey was completed by 395 participants, of whom 74.2% were female and approximately 59.3% aged <45 years. Responders reported having problems with mobility, pain, and fatigue with older participants reporting higher levels of pain and more mobility problems, although the correlations were fairly small. Healthcare usage was surprisingly low.

**Conclusion:**

The study found that age was associated with a decline in mobility and a higher level of pain, although the relationships were weak. It is possible that the low healthcare usage among the responders is owing to services not being available to respond to their needs.

## How this fits in

To the authors’ knowledge, there are no UK studies that have examined the effects of ageing on adults with cerebral palsy (CP). Building a knowledge base about the current healthcare experiences of adults with CP, and their changing health needs, is vital to enable health and rehabilitation practitioners to ensure the population receive well-coordinated long-term care. This study has shown that adults with CP experience problems with pain and mobility as they age, but healthcare usage is relatively low.

## Introduction

CP, one of the most common lifelong neurological impairments, results from a non-progressive injury in the fetal or infant brain either before, after, or during birth.^1^ It affects limbs and the motor system differently, depending on the part of the infant brain that has been damaged, and varies in terms of severity. International reporting on the prevalence of CP ranges from 1.5 to >4 per 1000 live births.^2–5^ In the UK, the National Institute for Health and Care Excellence (NICE) estimates 2–3.5 per 1000 live births result in CP.^6^ This stable prevalence, coupled with the fact that most people with CP have a full life expectancy, means that adults with CP are increasingly living into older adulthood.^7^ The prevalence of CP is estimated at 17 million worldwide.^8^

As people with CP age, they experience physiological changes at an accelerated rate when compared with their non-disabled peers,^9,10^ which differ to those changes experienced in childhood or adolescence.^11–13^ These changes include a deterioration in mobility, increases in pain and fatigue, and an increase in falls and fractures.^14,15^ Helping adults with CP stay healthy throughout the life course will have long-term cost benefits via reductions in hospital admissions, long-term care, and unemployment rates.

Although extensive specialist knowledge and rehabilitation is available for children with CP in the UK, people with CP report feeling abandoned by the adult healthcare system as their health needs (existing and new) are no longer being met.^13,15,16^ Owing to the lack of dedicated adult services for people with CP, some paediatricians keep their patients on their caseload beyond age 18. To the authors’ knowledge, there are no studies in the UK that have quantified different aspects of healthcare utilisation and embodied effects of CP across the life course. NICE has published guidelines (https://www.nice.org.uk/guidance/ng119) that start to address some of the issues in relation to how to improve the health, wellbeing, function, and participation of adults with CP. However, UK adult rehabilitation services for people with CP as they age are limited. Building a knowledge base about the current healthcare experiences of adults with CP, and their changing health needs, is vital to enable health and rehabilitation practitioners to ensure the population receives well-coordinated long-term care.

This article reports the results of a national online survey, which explored how adults with CP experience ageing. The purpose of the study was to examine the effects of growing older on adults with CP and their changing healthcare needs.

## Method

The study was designed by the research team with considerable input from five people with CP (with diverse age, background, gender, and type of CP).

### Participants

The participants were adults with CP in the UK, aged ≥18 years who had access to a computer to complete the online survey.

### Online survey

#### Item selection

The items for the survey were taken from self-report measures that were identified through a MEDLINE search, a Google and Google Scholar search, and looking through the reference lists of articles identified through the searches (snowballing). A total of 684 abstracts were identified in MEDLINE, six more through the Google Scholar search, and 13 more through snowballing. For the MEDLINE search, the search terms ‘cerebral palsy’ and ‘adults’ were used along with the domain being searched, such as ‘dexterity’. The domains were mobility, dexterity, fatigue, pain, speech, and mental health. If the search did not identify any articles, the search was expanded by substituting ‘disabled’ or ‘disabled persons’ for ‘cerebral palsy’. The searches had the limiters ‘humans’ and ‘English language’, and were run between 26 August 2021 and 24 September 2021. Both a keyword search and the ‘Map Term to Subject Heading’ option were used. The swallowing measure that was used was found through a Google Scholar search and the health maintenance measure that was used through snowballing; the others were found through the MEDLINE search.

Table 1 shows the domains and the instruments from which the items were drawn. The authors did not want to burden participants with a very long questionnaire but wanted to sample all of the above-mentioned domains. Therefore, for some items, the entire scale was not used and items most relevant for people with CP were selected following discussion within the team.

**Table 1.** Domains of interest and where items were found

Domain	Where items were found	Full scale used?
Mobility	Mobility subscale of WHO Disability Assessment Schedule (WHODAS 2.0)^26^	Yes
Dexterity	3 items from Arthritis Impact Measurement Scales 2-Short Form^27^	No
Fatigue	Fatigue Severity Scale short form^28^ ^,a^	Yes
Pain	Pain portion of Short Form (SF)-36 questionnaire^23^	Yes
Speech	3 items from the Living With Neurologically Based Speech Difficulties^29^	No
Mental health	WHO-5 Well-Being Index^30^	Yes
Health maintenance/self-care	Health Confidence Score^31^	Yes
Swallowing	Pharyngeal symptoms from swallowing-quality of life (SWAL-QOL) questionnaire^21^	No

^a^An item was added to this scale concerning the effects of fatigue on speech.

Additional questions were included about fatigue, pain, and swallowing, which are listed in the Results section. A series of demographic questions were included at the end of the survey to document participants’ ethnic group, gender, sexual orientation, and age. A further series of questions were included about type of CP, with four options: ataxic, dyskinetic, spastic, or mixed. Participants who chose the spastic CP option navigated to a further question about the type of spastic CP they had: hemiplegia, diplegia, triplegia, or quadriplegia. There was also a section of the survey that asked about healthcare usage.

#### Procedure

The items were put into Jisc online surveys (https://www.onlinesurveys.ac.uk/) along with demographic questions as well as questions concerning healthcare usage. The survey was first pilot tested, via the online platform Zoom, with 10 adults with different types of CP, based across the UK. The aim was to ensure that the wording and response options were appropriate and accessible to survey participants. Input was also obtained from the Patient and Public Involvement (PPI) group, which was made up of five individuals living with CP who met regularly online during the project. This group played an integral role in the framing of the questions and design of the survey using both lived and professional experience, which ensured that relevant questions were asked and were framed in an accessible, understandable way.

The think-aloud technique^17^ encouraged participants to verbalise their thoughts as they completed the survey, especially any changes that they thought needed to be made. Each think-aloud Zoom session was recorded and transcribed. Transcriptions were analysed, and changes were discussed with the research team and made to the online survey. The final online survey was launched in March 2022 for Cerebral Palsy Awareness Month and was circulated through various channels, including the University of Nottingham and Scope (the disability equality charity https://www.scope.org.uk/) press teams, the project team and their networks in disability studies, rehabilitation, and health sciences. Scope posted the link to its online community, and sent the survey to other disability agencies across the UK, to the CP All Party Parliamentary Group, and to partners of the CP consortium. Scope shared the survey via its targeted Twitter (now known as X) and Instagram channels, repeating this in April and June 2022. The link was also posted on the following Facebook groups for adults with CP: Adult CP UK; Women Ageing with CP; and Adult CP Hub. The survey was open between 4 March 2022 and 20 July 2022.

The data were analysed using IBM SPSS Statistics for (version 28).^18^ Frequency counts for the demographic variables and the survey questions were examined. Descriptive statistics were calculated for the survey variables, with means and standard deviations used for interval-level variables and medians and interquartile ranges used for ordinal-level variables. Finally, crosstabs were run to examine the relationships between the demographic variables (age, gender, and type of CP) and the survey responses. Chi-square was calculated, with the Phi coefficient used to measure the level of association.

## Results

The survey was completed by 395 participants. More than 90% (92.9%, *n* = 367) of the responders who completed the questionnaire were people with CP; 7.1% (*n* = 28) of the questionnaires were completed by a proxy. As shown in Table 2, most of the participants were White (92.4%, *n* = 365) and female (74.2%, *n* = 293), with more than half (52.2%, *n* = 206) being in either the 25–34 years or 35–44 years age groups. The majority had spastic CP (63.8%, *n* = 252). Among those with spastic CP, 41.7% (*n* = 105) had diplegia, with the rest having hemiplegia (30.6%, *n* = 77), triplegia (3.6%, *n* = 9), or quadriplegia (24.2%, *n* = 61).

**Table 2.** Demographic characteristics

Question	Frequencies and proportions, *n* (%)	
1) How would you describe your ethnicity or ethnic background?	Arab	1 (0.3%)
	Asian	9 (2.3%)
	Black	9 (2.3%)
	Mixed	8 (2.0%)
	White	365 (92.4%)
	Prefer not to say	3 (0.8%)
2) How would you describe your gender?	Male	98 (24.8%)
	Non-binary	3 (0.8%)
	Female	293 (74.2%)
	Any other gender	1 (0.3%)
3) How would you describe your sexual orientation?	Asexual	13 (3.3%)
	Bisexual	32 (8.1%)
	Gay or Lesbian	14 (3.5%)
	Heterosexual	308 (78.0)
	Queer	6 (1.5%)
	Prefer not to say	21 (5.3%)
	Any other	1 (0.3%)
4) What type of CP do you have?	Ataxic	18 (4.6%)
	Dyskinetic	27 (6.8%)
	Mixed	81 (20.5%)
	Spastic	252 (63.8%)
	Not sure or prefer not to say	17 (4.3%)
5) How old are you?	18–24 years	28 (7.1%)
	25–34 years	103 (26.1%)
	35–44 years	103 (26.1%)
	45–54 years	74 (18.7%)
	55–64 years	64 (16.2%)
	≥65 years	22 (5.6%)
	Prefer not to say	1 (0.3%)
6) What type of spastic CP do you have?	Diplegia (two lower limbs affected)	105 (41.7%)
	Hemiplegia (one side of the body affected)	77 (30.6%)
	Triplegia (three limbs affected)	9 (3.6%)
	Quadriplegia (four limbs affected)	61 (24.2%)

CP = cerebral palsy.

The descriptive statistics for the items that comprised the survey can be found in Supplementary Table S1 with the frequencies and percentages for these items in Supplementary Table S2. There are several values of note in Supplementary Table S1. First, responders reported that they experienced severe or extreme problems with standing for long periods and walking a long distance. Most (65.1%) had avoided activities owing to a fear of falling and had fallen more than three times in the past 4 weeks. As shown in Supplementary Table S1, adults tended to agree that fatigue caused frequent problems for them, prevented sustained physical functioning, and interfered with carrying out duties and responsibilities. Participants chose to describe their level of bodily pain as ‘moderate’ and almost 10% had received medical treatment for aspiration of food or drink. Regarding how they felt, responders tended (median of 5 on a 6-point scale) to disagree with the statement that they ‘*woke up feeling fresh and rested*’.

The relationships between the survey questions in Supplementary Table S1 and the demographic variables in Table 2 were examined. First, crosstabs were run between gender, age, and type of CP and the questions in italics from Supplementary Table S1. These questions were chosen because the descriptive statistics suggested that responders had particular difficulties in these areas; the descriptive statistics for these items by age group can be found in Supplementary Table S3. With the exception of contacts with GPs and the use of medication or assistive devices, investigating the relationships between the demographic variables and healthcare usage was not possible owing to the small number of people in each category. The survey questions and demographic variables were coded as dichotomous variables (with the exception of age, which was split into three groups) to ensure an adequate cell size. The coding can be seen in Supplementary Table S2.

Owing to the large sample size, many relationships were significant, so the study initially only looked at correlations where the Phi coefficient was 0.20 or greater, which indicates at least a weak association. For age, gender, and type of CP (spastic or not), the only moderately weak relationships were found for age and difficulty of standing up from sitting down (Phi = 0.20, *P*<0.01), gender and bodily pain (Phi = -0.20, *P*<0.01), and for type of CP and coughing (Phi = 0.25, *P*<0.01). People who were older reported more problems with standing up from sitting down (48.1% in the 18–34 year group as opposed to 62.1% in the 35–54 year group, and 77.4% in the >55 year group), women reported more bodily pain than men (72.7% versus 51.0%); and those with spastic CP reported fewer problems with coughing (40.9% versus 67.5%).

However, given the main purpose of the study, we decided to examine the relationships between age and the outcome variables more closely. As Table 3 shows, age was significantly correlated with a decline in mobility, increase in pain, and difficulty in swallowing. Among the mobility items, a higher percentage of older people (those aged ≥55 years) compared with younger people (18–34 years) tended to avoid activities owing to a fear of falling, had difficulty walking a long distance, had difficulty standing up from sitting down, avoided lifting and carrying objects, avoided walking on different surfaces, and walked with an aid. A higher percentage of older people had at least a moderate level of bodily pain, experienced pain that interfered with work, and had problems with coughing.

**Table 3.** Associations between age and outcomes

Age, years (18–34, 35–54, ≥55)	χ^2^,significance level,Phi coefficient	Percentages
*Mobility*		
I avoid activities due to a fear of falling	χ^2^ = 8.1, *P*<0.05Phi = 0.14	55.7% (18–34) *n* = 7368.9% (35–54) *n* = 12272.1% (≥55) *n* = 62
Difficulty walking a long distance, such as a mile	χ^2^ = 8.2, *P*<0.05Phi = 0.14	61.1% (18–34) *n* = 8070.6% (35–54) *n* = 12579.1% (≥55) *n* = 68
Difficulty standing up from sitting down	χ^2^ = 15.5, *P*<0.01Phi =0.20	48.1% (18–34) *n* = 6362.1% (35–54) *n* = 11074.4% (≥55) *n* = 64
Due to my fear of falling, I avoid lifting and carrying objects	χ^2^ = 9.5, *P*<0.01Phi = 0.15	29.8% (18–34) *n* = 3946.9% (35–54) *n* = 8343.0% (≥55) *n* = 37
Due to my fear of falling, I avoid walking on different surfaces	χ^2^ = 8.0, *P*<0.05Phi = 0.14	37.4% (18–34) *n* = 4953.7% (35–54) *n* = 9546.5% (≥55) *n* = 40
Do you walk with an aid? (walking stick, walker or wheelchair)	χ^2^ = 7.6, *P*<0.05Phi = 0.14	44.3% (18–34) *n* = 5848.0% (35–54) *n* = 8562.8% (≥55) *n* = 54
*Pain*		
My level of bodily pain is moderate or severe	χ^2^ = 6.3, *P*<0.05Phi = 0.13	59.5% (18–34) *n* = 7868.4% (35–54) *n* = 12175.6% (≥55) *n* = 65
Pain interferes with my work (either outside the home or housework)	χ^2^ = 8.1, *P*<0.05Phi = 0.15	59.1% (18–34) *n* = 7573.3% (35–54) *n* = 12173.8% (≥55) *n* = 62
*Swallowing*		
Problems with coughing	χ^2^ = 7.5, *P*<0.05Phi = 0.14	41.2% (18–34) *n* = 5452.5% (35–54) *n* = 9359.3% (≥55) *n* = 51

Totals for age groups: 18–34 years *n* = 131; 35–54 years *n* = 177; ≥55 years *n* = 86

## Discussion

### Summary

The findings indicated that, for adults with CP there is an association between ageing, a decline in mobility, and a higher level of pain, although the relationships were weak. Women reported more bodily pain than men. The responders were predominately White (92.4%) and female (74.2%)

### Strengths and limitations

One strength of this study was that it included a large number of responders (*n* = 395) throughout the UK using an online approach, which allowed people to participate from their homes using their own technology. Of course, this required that people had an online presence, which meant that some people will have been excluded from the sample, especially those with lower socioeconomic status, which the study did not measure. The sample studied was recruited from several sources including Scope, various Facebook groups, Twitter (now known as X), and other disability agencies across the UK. While there were advantages to this in terms of reaching different groups of people with CP, a disadvantage is that it is not known how many people received information about the survey and a response rate cannot be calculated. Another limitation is that the sample was predominately White and female, and included relatively few people aged ≥65 years; this limits the generalisability of the findings. Also, the geographical spread and educational attainment of responders were not known.

The study sampled items from several domains of concern for people with CP including mobility, dexterity, fatigue, pain, speech, swallowing, feelings, health maintenance, and healthcare usage. We were guided in this endeavour by researchers who have CP, a PPI group of adults with CP, and pilot tests with people who have CP, who could help pinpoint the areas of concern and refine the questions. Adopting this approach meant some scales needed adapting as nothing similar existed.

An assumption could be made that the sample appeared to be relatively healthy based on their low healthcare usage. However, given the problems experienced by people with CP in the sample, this would be surprising. The small number of people in each category made it difficult to explore correlations between the demographic variables and healthcare usage (except GP contacts and the use of medication or assistive devices). Given the literature and hints from the survey data, we believe that the low healthcare usage among the responders could be owing to services not being available to respond to their needs. In this regard, it should be noted that for the responders, contacts with GPs were below the national average, with an average of 6.3 contacts per person per year, whereas the average number of contacts for GPs in the UK was 8.3 per person per year.^19^

### Comparison with existing literature

The present study has found that people with CP have more problems with pain and mobility as they age, although it should be noted that the associations were weak. Benner *et al* ^20^ also found that pain increased and mobility decreased over a 14-year period for people with CP in Rotterdam, but the age of the oldest participants in their sample was only 45 years, whereas the present sample contained many participants (40.5%) aged >45 years; however, the percentage of participants in the present study who had spastic CP (63.8%) was similar to the percentage reported by Benner (75%).

In comparing the sample with other studies that have looked at people with CP, it was found that fewer people in the present sample reported problems with swallowing, but more problems with pain. On most of the swallowing measures, Yi *et al’*s^21^ responders in Korea were 2–3 times more likely to have difficulties, but this may have been due to the high percentage of people with quadriplegia in their sample (73.5%). However, a larger percentage of the present sample reported ‘severe’ or ‘very severe’ levels of bodily pain than either Jahnsen *et al* ^22^ or Rodby-Bousquet *et al*,^23^ and a larger percentage reported that pain interfered ‘quite a bit’ or ‘extremely’ with work outside the home or housework. Our sample had a higher percentage of female participants than either of these studies, which may have led to these findings, although Rodby-Bousquet *et al* ^23^ found that females reported more bodily pain as did the present study. More generally, the literature has suggested that women are more sensitive to pain than men,^24,25^ although to what extent this is cultural is unclear.^25^

### Implications for practice and research

The study confirms that people with CP in the UK have considerable problems with mobility, balance, falls, fatigue, pain, and report more difficulties as they age. Low usage of health services may be due to a paucity of adult specialist services. Further research could help with the development and evaluation of potential improvements to services.

Paediatric and adult services for people with CP need to be better integrated so that services can anticipate the needs of those with CP as they reach adulthood. Showcasing GP practices that are doing well would also set an example for those practices that are trying to improve their services for people with CP. To gain greater access to GPs, people with CP may deliver training to GP trainees so that they are better educated about the issues that people with CP experience.

## Data

The dataset relied on in this article is available from AJA on reasonable request (Anthony.Avery@nottingham.ac.uk).
